# High-Efficiency Reverse (5′→3′) Synthesis of Complex DNA Microarrays

**DOI:** 10.1038/s41598-018-33311-3

**Published:** 2018-10-10

**Authors:** Kathrin Hölz, Julia K. Hoi, Erika Schaudy, Veronika Somoza, Jory Lietard, Mark M. Somoza

**Affiliations:** 1 Institute of Inorganic Chemistry, Faculty of Chemistry, University of Vienna, Vienna, Austria; 2 Department of Physiological Chemistry, Christian Doppler Laboratory for Bioactive Aroma Compounds, Faculty of Chemistry, University of Vienna, Vienna, Austria

## Abstract

DNA microarrays are important analytical tools in genetics and have recently found multiple new biotechnological roles in applications requiring free 3′ terminal hydroxyl groups, particularly as a starting point for enzymatic extension via DNA or RNA polymerases. Here we demonstrate the highly efficient reverse synthesis of complex DNA arrays using a photolithographic approach. The method is analogous to conventional solid phase synthesis but makes use of phosphoramidites with the benzoyl-2-(2-nitrophenyl)-propoxycarbonyl (BzNPPOC) photolabile protecting group on the 3′-hydroxyl group. The use of BzNPPOC, with more than twice the photolytic efficiency of the 2-(2-nitrophenyl)-propoxycarbonyl (NPPOC) previously used for 5′→3′ synthesis, combined with additional optimizations to the coupling and oxidation reactions results in an approximately 3-fold improvement in the reverse synthesis efficiency of complex arrays of DNA oligonucleotides. The coupling efficiencies of the reverse phosphoramidites are as good as those of regular phosphoramidites, resulting in comparable yields. Microarrays of DNA surface tethered on the 5′ end and with free 3′ hydroxyl termini can be synthesized quickly and with similarly high stepwise coupling efficiency as microarrays using conventional 3′→5′ synthesis.

## Introduction

Since their development, DNA microarrays have had a high impact in biology and medicine and have become widely used, powerful analytical tools in a variety of applications^1,2^, including gene expression profiling^3–5^, genotyping^6–10^ and resequencing^11–13^. The fabrication of DNA microarrays can be accomplished *via* multiple methods, for example by mechanically spotting purified DNA oligonucleotides onto a solid surface or by *in situ* DNA synthesis by ink-jet-like printing of activated phosphoramidites^14^. Photolithography, the original approach to *in situ* synthesis, allows for the fabrication of high-density microarrays using near ultraviolet light as the 5′-OH deprotection trigger^15,16^. Further refinement of the technology led to the Maskless Array Synthesis (MAS) system, which allows for the use of virtual masks *in lieu of* physical lithography masks. MAS photolithography is a flexible and robust method that can achieve high complexity and high density, not only in DNA synthesis^4,17^, but also in RNA synthesis^18,19^, peptide synthesis^20,21^ and for the synthesis of microarrays of biopolymer mimics with engineered properties^22,23^. Like conventional solid phase synthesis of oligonucleotides, microarray synthesis is almost always performed in the 3′ to 5′ direction; however, promising new applications based on enzymatic processing require a free 3′ end and thus need reverse synthesis. Examples of these applications include genotyping by allele-specific primer extension^8–10^ and spatial transcriptomics technology, which combines gene expression analysis and visualization of tissue slices while retaining morphological context^3,24,25^. Mechanically spotted DNA arrays are well suited for those experiments, since oligonucleotides are typically attached to the surface using 5′ amino or biotin modifications. Spotting has the additional advantage that synthetic, pre-purified oligonucleotides as well as significantly longer DNA strands, such as PCR products, can be attached to the microarray surface^10,26^. While spotting may overcome the length and directionality limitations of *in situ* array synthesis, spot homogeneity and spot density are severely restricted in comparison with photolithographic synthesis.

Light-directed MAS requires photolabile protecting groups on nucleoside phosphoramidites in order to carry out the controlled synthesis of oligonucleotides^27^. Early examples of photoprotected phosphoramidites featured a nitrophenyl core as the photosensitive moiety (NVOC^28^, MeNPOC^29^). Attaching an ethyl group *meta* to the nitro substituent resulted in a very significant increase in photolysis rate^30^, leading to the development of NPPOC (2-(2-nitrophenyl)-propoxycarbonyl)^29^, which Beier and Hoheisel adapted for phosphoramidites and used in the light-directed, *in situ* fabrication of DNA microarrays^30^. Recent work on improving the photocleavage efficiency of the NPPOC group culminated with the preparation of benzoyl and thiophenyl-substituted NPPOC (BzNPPOC and SPhNPPOC, respectively), which have photolytic efficiencies up to 12 times higher than standard NPPOC, thereby greatly reducing the overall array synthesis time^31,32^.

To broaden the spectrum of applications of high-density photolithographic DNA microarrays by allowing the elongation of oligonucleotides in the 5′→3′ direction, phosphoramidites equipped with an NPPOC group at the 3′-OH position have been synthesized^33^. While the 5′-hydroxyl group is known to be more reactive than the less nucleophilic 3′-OH group, the stepwise addition of 3′-NPPOC phosphoramidite monomers resulted in high coupling yields^34^ and thus allowed for their use in the photolithographic *in situ* synthesis of reverse DNA microarrays. Complex microarrays containing more than 150000 probe sequences were fabricated with reverse, 3′-NPPOC 5′-amidites and the corresponding 5′→3′ oriented strands were found to be accessible substrates for various enzymatic processing reactions^34^.

**Figure 1:**
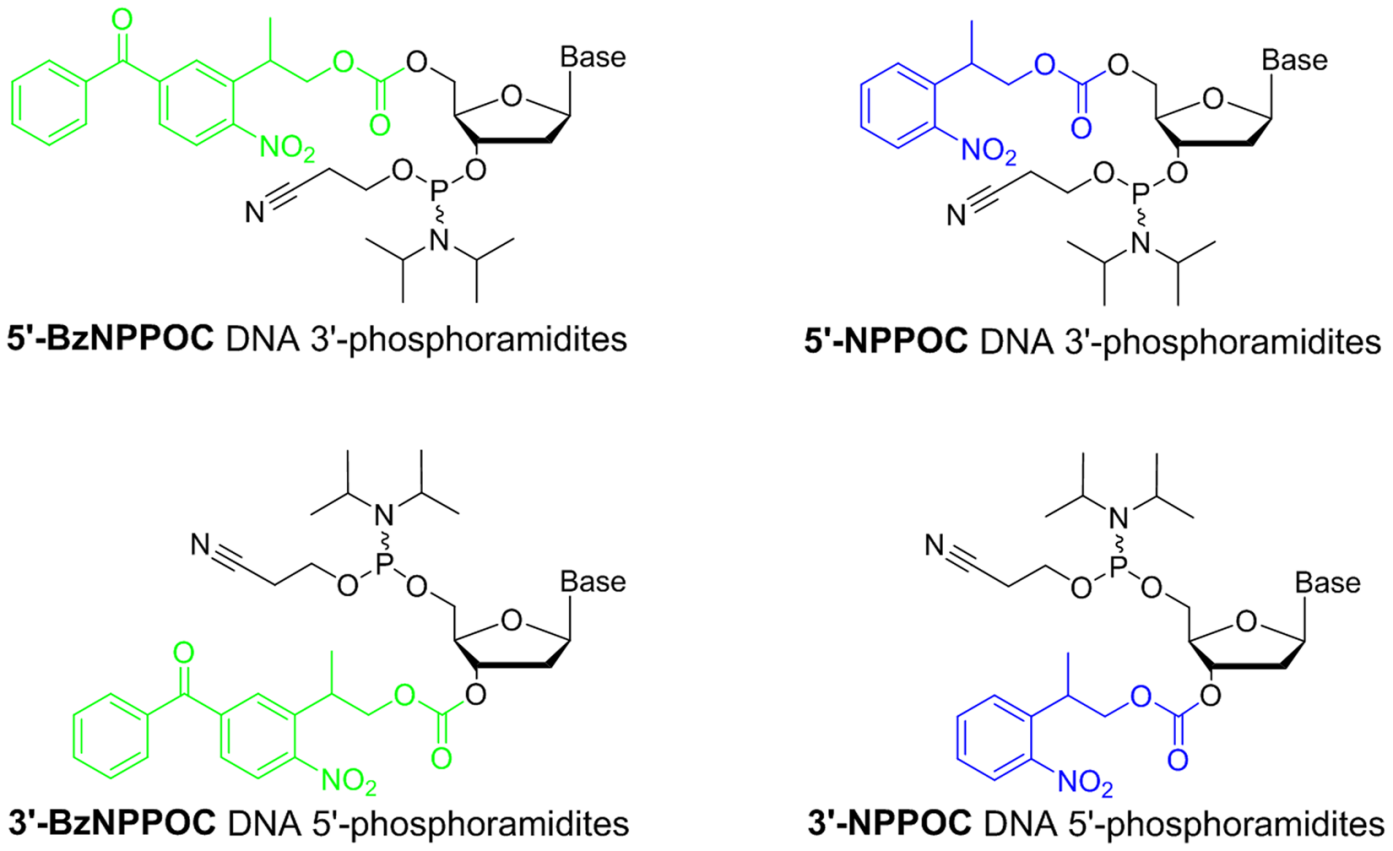
Chemical structures of DNA phosphoramidites with NPPOC and BzNPPOC 3′- or 5′-OH protecting groups.

While the development of light-directed reverse array synthesis opened up the way to applications requiring enzymatic interactions, we hypothesized that the relatively long synthesis time of microarrays with reverse, NPPOC-protected monomers could lead to significant limitations. Indeed, in previous work, we showed that a long and repeated exposure to the reagents and solvents used during the synthesis slowly degrades the microarray surface and therefore decreases chip quality^31^. Hence, short synthesis times are preferable in order to hinder the degradation of the surface, which in turn poses a limit to oligonucleotide length. Here we report on the use of 3′-benzoyl-2-(2-nitrophenyl)-propoxycarbonyl (BzNPPOC) as an alternative, high-efficient 3′-photolabile protecting group as well as on improvements in the fabrication process of DNA arrays of reverse orientation (Fig. 1). The experiments presented here include the evaluation of coupling and photolysis efficiency as well as the determination of the optimal coupling and deprotection times. Those advancements have allowed for a fast synthesis of complex high-density 5′→3′ DNA oligonucleotide microarrays of increased length, and with the same overall synthesis efficiency as 3′→5′. This progress now offers high-resolution and high-throughput platforms for DNA microarray assays with enzymatic processing.

## Results

### Coupling efficiency

**Figure 2:**
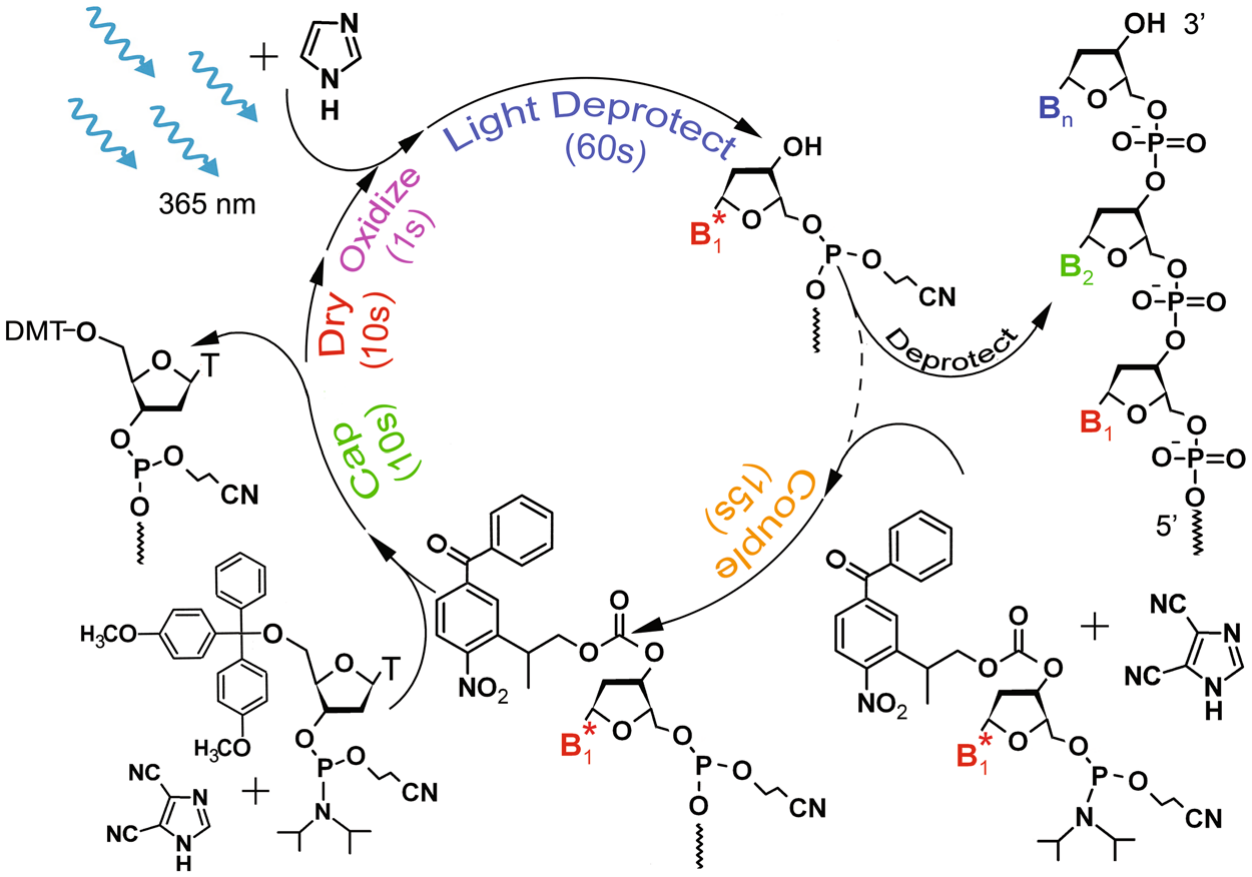
Representative synthesis cycle in maskless, light-directed synthesis of reverse microarrays using 3′-BzNPPOC phosphoramidites. A high-power UV LED is used to photodeprotect the 3′-OH in the presence of a weak organic base. The final chemical deprotection removes the cyanoethyl and base protecting groups. An optional capping step with 5′-DMTr-dT caps any remaining unreacted 3′-hydroxyl groups. Representative step times are given but depend on specific experimental parameters and objectives.

Commercially available 3′-NPPOC and 3′-BzNPPOC protected phosphoramidites were used to determine the stepwise coupling yield of each monomer in reverse, *in situ* DNA microarray synthesis. A schematic illustration of a synthesis cycle with 3′-BzNPPOC monomers is shown in Fig. 2. To determine the stepwise coupling yield, oligonucleotides of various chain lengths were terminally-labelled with a fluorescent dye, as previously described^35^. In order to terminate the elongation of an oligonucleotide after failed monomer incorporations, the coupling step was followed by a capping event. Although different capping agents can be used for this purpose, e.g. acetic anhydride or “Unicap” (diethyleneglycol ethyl ether (2-cyanoethyl)-(*N*,*N*-diisopropyl)-phosphoramidite), 5′-DMTr-dT essentially acts as a capping agent as well since photolithography bypasses the use of an acidic deblocking step. With a high coupling efficiency, 5′-DMTr dT was preferred over standard capping mixtures^35,36^. The final step consisted in adding a Cy3 dye at the 3′ end of the oligonucleotide in 50% of the synthesized features. The remaining features were deliberately not labelled and served as a reference for background fluorescence. According to this method, the coupling efficiencies for the stepwise addition of NPPOC and BzNPPOC phosphoramidites were determined for synthesis in the 3′→5′ as well as in the 5′→3′ direction and are shown in Table 1.

**Table 1:** Coupling efficiencies of DNA phosphoramidites. *Left*.

Phosphoramidites	Coupling efficiency (%)	Phosphoramidites	Coupling efficiency (%)
**5**′ **NPPOC**		**5**′ **BzNPPOC**	
dA	99.9	dA	99.9
dC	99.3	dC	99.9
dG	97.4	dG	97.1
dT	99.9	dT	99.9
**3**′ **NPPOC**		**3**′ **BzNPPOC**	
dA	99.9	dA	99.4
dC	99.0	dC	98.8
dG	93.9	dG	97.6
dT	99.9	dT	99.9

Stepwise coupling yield for monomers carrying a NPPOC photolabile protecting group on either the 5′- or the 3′-OH. *Right*. Stepwise coupling yield for monomers carrying a BzNPPOC protecting group on either the 5′- or the 3′-OH. The coupling time per synthesis cycle was set to 60 seconds.

Previous coupling experiments performed by Pirrung *et al*. showed that commercial 3′-DMTr phosphoramidite monomers required increased coupling time compared to their 5′-DMTr variants in order to reach similar coupling efficiencies^33^. Comparable results were also obtained for the stepwise coupling of 3′-NPPOC monomers^34^. The lower coupling efficiency of 5′ phosphoramidites may be attributable to the lower nucleophilicity of the 3′-hydroxyl group compared to the more reactive 5′-hydroxyl group. In our hands, we were able to obtain coupling yields equal or superior to 99% for reverse dA, dC and dT monomers and sub 98% for dG, within the same range as those of 5′-NPPOC and 5′-BzNPPOC monomers in standard 3′→5′ oligonucleotide synthesis.

### Photolysis efficiency

**Figure 3:**
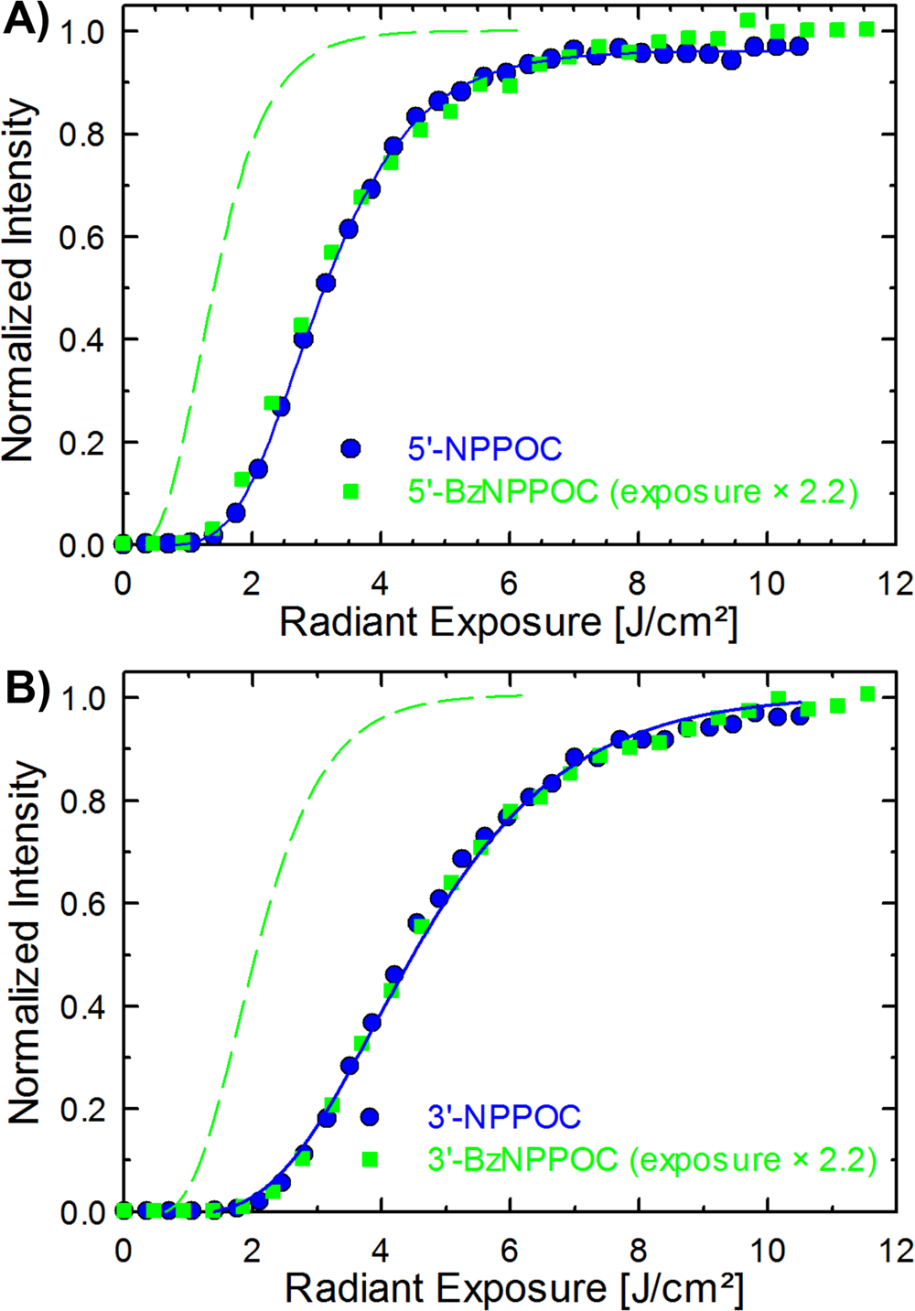
Hybridization-based fluorescence intensities for 25mer microarrays synthesized using a gradient of UV light. The microarrays were synthesized with either NPPOC or BzNPPOC phosphoramidites. (**A**) 3′→5′ synthesis with 5′-NPPOC and 5′-BzNPPOC monomers. (**B**) 5′→3′ synthesis with 3′-NPPOC and 3′-BzNPPOC monomers. In both graphs, the radiant exposure values for the BzNPPOC syntheses are multiplied by 2.2, with original data positions indicated by the dashed lines. For both NPPOC and BzNPPOC, 3′ photolysis requires 45% more light than 5′ photolysis.

The photolabile NPPOC and BzNPPOC protecting groups show very similar absorbance in the spectral region near 365 nm and are now commonly used in the light-directed maskless synthesis of microarrays. The BzNPPOC group offers a sustainably faster photolysis rate over the standard NPPOC group due to its increased photolytic efficiency, which translates into much faster photocleavage than for the NPPOC group^32^. To determine the optimal light exposure parameters for removing the photolabile protecting groups of the corresponding 3′-OH protected monomers, light exposure gradient experiments were performed. After synthesizing 25mer DNA strands on microarrays using a gradient of UV light exposures, the oligonucleotides were hybridized to their Cy3-labelled complementary strand. The normalized fluorescence intensities for syntheses performed in the forward as well as the reverse direction with either NPPOC or BzNPPOC monomers are shown in Fig. 3. The shape of the curves shows an increase in hybridization signals with increasing UV light exposure that eventually nears saturation. This rise corresponds to an increase in sequence fidelity. Indeed, an incomplete removal of the photolabile protecting group introduces errors in the sequence, resulting in weaker hybridization efficiency and, in turn, lower fluorescence intensity. In order to make direct comparisons of the hybridization intensities obtained with BzNPPOC and NPPOC monomers, the curves were superimposed by scaling the radiant exposure values for BzNPPOC by 2.2.

The resulting overlapping curves can be understood as photolysis kinetics and thus indicate a photolysis rate for BzNPPOC more than twice as fast as that of NPPOC. Indeed, 95% photocleavage of the 5′-NPPOC group can be achieved with an exposure of 60 s at 100 mW/cm^2^, for a total radiant exposure of 6 J/cm^2^, while the equivalent removal of the 5′-BzNPPOC group requires under 30 s, or 3 J/cm^2^. Comparing the photolysis efficiency of 3′- with 5′-protected amidites with a photosensitive protecting group of the same series reveals a higher photocleavage rate for the 5′-protected monomers than for their 3′-protected counterparts, for both NPPOC and BzNPPOC.

### Deprotection time optimization

**Figure 4:**
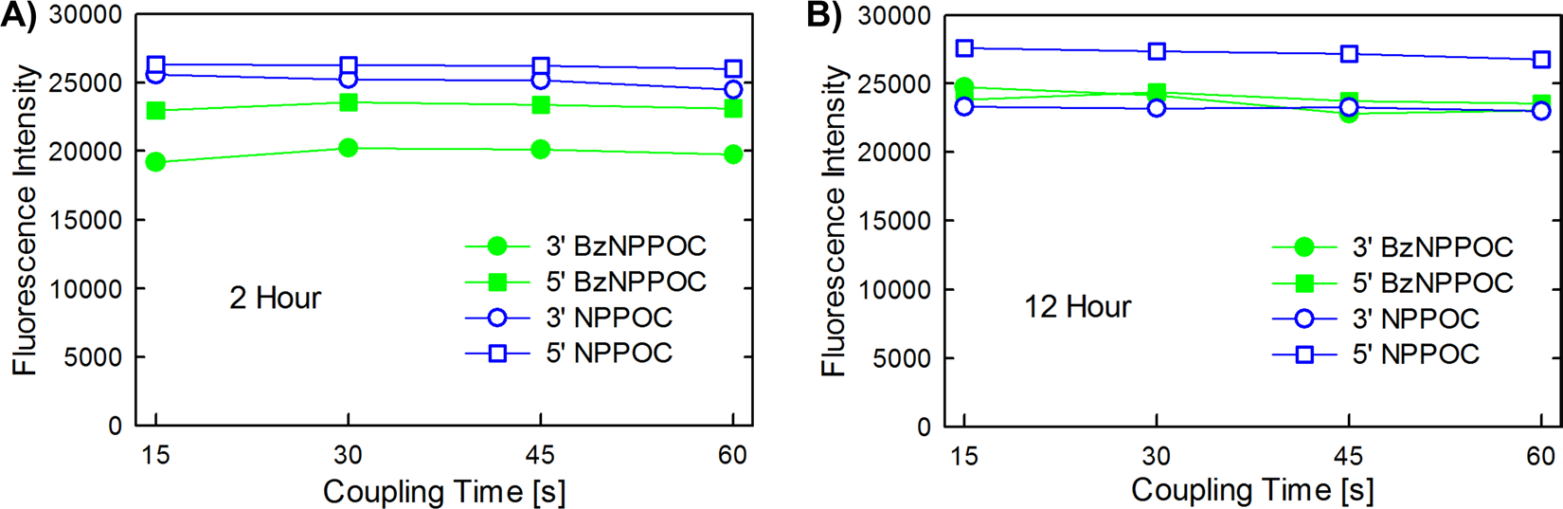
Investigation of the optimal coupling and deprotection times for 3′- and 5′-NPPOC and BzNPPOC protected phosphoramidites. All data points correspond to the synthesis of the same mixed-base oligonucleotide 25mer. Coupling times were 15, 30, 45 or 60 seconds. The deprotection of the nucleobase and phosphate protecting groups was performed in 1:1 (v/v) EDA/EtOH for either 2 hours (**A**) or 12 hours (**B**).

The chemical deprotection at the end of a synthesis removes the protecting groups of the phosphodiester moiety and of the exocyclic amine of the nucleobase. For microarrays, the deprotection usually takes place in a 1:1 (v/v) solution of ethylenediamine and ethanol and is complete within 2 hours for monomers carrying fast-deprotecting groups from the phenoxyacetyl series (e.g. Pac, *t*BPac and *i*PrPac) at the nucleobase, while phosphoramidites with more stable base protecting groups require an extended treatment in the deprotection solution. In order to define the minimum time period for complete deprotection, mirror-image array pairs originating from the same synthesis were exposed to ethylenediamine/ethanol for either 2 or 12 hours. The optimal settings can be determined by hybridizing Cy3-labelled complementary oligonucleotides to the DNA strands synthesized on the microarray surface. Indeed, partially deprotected nucleobases are expected to reduce hybridization to the complementary strand because the protected exocyclic amines of A, C and G cannot form hydrogen bonds and because the protecting groups may destabilize the base stack via steric hindrance. This effect can be seen in lower Cy3 fluorescence intensity after hybridization following incomplete deprotection. Figure 4 shows the hybridization results of the synthesized 25mer for both deprotection periods. Whereas the oligonucleotides fabricated with 3′-NPPOC, 5′-NPPOC and 5′-BzNPPOC phosphoramidites are fully deprotected after 2 hours, those made with 3′-BzNPPOC phosphoramidites require an extended deprotection to reach completion due to the presence of a conventional benzoyl protecting group on the adenine base. The curves suggest that the deprotection can be performed over a period of 12 hours without any degradation of the oligonucleotides or the microarray surface.

### Coupling time optimization

The coupling of a phosphoramidite to a growing oligonucleotide chain is made possible after photodeprotection of the 3′-OH, in reverse synthesis. Previously, we set the coupling times to 15 seconds in 3′-to-5′-directed syntheses^31^. The overall synthesis time becomes a significant hurdle to synthesis throughput as the complexity and the length of the synthesized probes increase. In order to expedite the fabrication of complex reverse arrays, we examined whether coupling times shorter than the 60 seconds used to determine the coupling efficiency would result in products of comparable quality. To determine the optimal coupling time, we synthesized four sets of 25mer probes of the same sequence in series. The first set of probes was synthesized with a coupling time of 60 seconds and the other sets with successively decreasing coupling times (45, 30 or 15 seconds). In order to take into account the minor degradation of oligonucleotides after repeated exposure to synthesis reagents and solvents, as previously observed^31^, the four sets of probes were also synthesized in the reverse order in a separate array, starting with 15 seconds of coupling time, then 30, 45 and 60 seconds. The arrays were then hybridized to their fluorescently labelled complement and scanned. The combined average intensity values for each set of microarray syntheses are shown in Fig. 4. We were able to obtain consistently high hybridization intensities for the oligonucleotides synthesized with the shortest coupling time, 15 seconds, for all tested phosphoramidites and for both deprotection times (2 and 12 hours, see Fig. 4).

### Forward vs reverse synthesis

**Figure 5:**
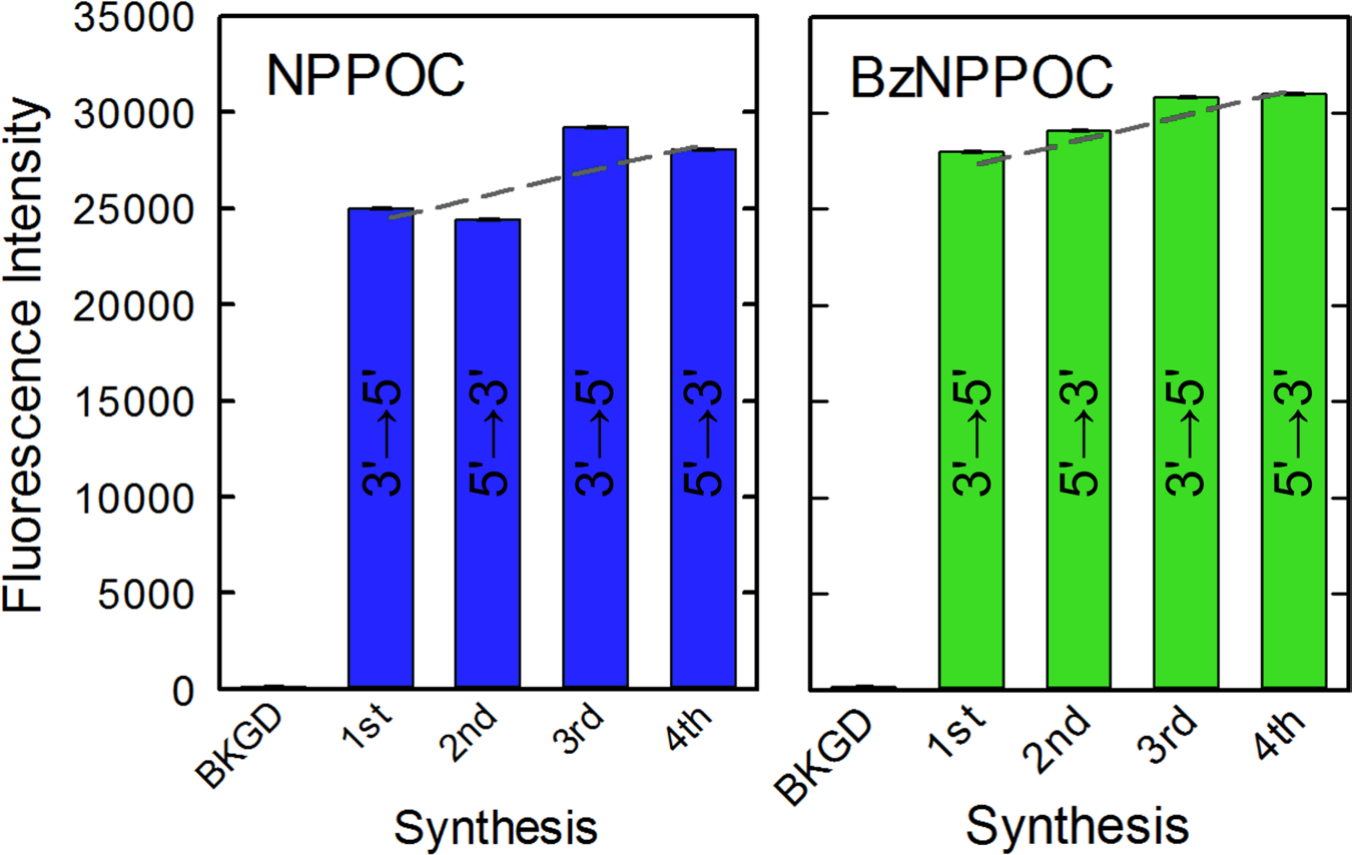
Fluorescence intensities from hybridized forward (3′→5′) and reverse (5′→3′) DNA strands synthesized consecutively on the same substrate. The strands were either synthesized using 3′- and 5′-NPPOC phosphoramidites or 3′- and 5′-BzNPPOC monomers. Hybridization was performed with an equimolar mix of 5′- and 3′-Cy3-labelled complementary oligonucleotides. Error bars are the SEM. The dashed lines indicate the expected ~6% trend in hybridization intensities for consecutively synthesized oligonucleotide 25mers.

We wished to combine the multiple optimization phases described above into a new and reliable protocol for the synthesis of reverse oriented DNA strands on microarrays, which could ideally be tested against the protocol for standard, 3′-to-5′-oriented DNA synthesis. In order to be able to make direct comparisons between the quality of the strands synthesized either in the 3′→5′ or 5′→3′ direction, forward and reverse synthesis must be performed on the same substrate to avoid variability inherent in hybridizing and washing separate microarrays. However, to prevent any accidental incorporation of phosphoramidites into a strand of wrong directionality, the microarray was designed with consecutive, rather than parallel, synthesis of forward and reverse sequences. The consecutive synthesis started with the fabrication of a 25mer sequence in the forward direction, followed by the synthesis of the same sequence in the reverse direction; this process order was repeated a second time on the same array. After deprotection, hybridization was performed with an equimolar mix of complementary oligonucleotides with a Cy3 dye either at the 5′ or the 3′ end. The use of an equimolar mix mitigates fluorescence intensity artefacts apparently related to the distance between the dye and the surface^37^. The complementary sequence terminated with Cy3 at the 3′ end had the final cytidine base swapped for a guanine, in order to maintain the identity of the nucleotide directly adjacent to the cyanine dye and prevent any sequence-dependent distortion of the fluorescence intensity^38,39^. The recorded fluorescence intensities resulting from hybridization to the DNA strands are shown in Fig. 5. Similar intensity values indicate successful syntheses of both forward and reverse oriented DNA strands. In sequential synthesis, the oligonucleotides synthesized earlier result in lower hybridization intensity due to chemical damage resulting from prolonged exposure to solvents and reagents. The difference in intensity is consistent and amounts to ~6% per consecutive 25mer synthesis of probe sets^31^. The expected trend is shown in Fig. 5 as dashed lines.

### Gene expression microarrays

The next step was to apply our newly established synthesis protocol to the fabrication of highly complex and demanding microarrays. A typical use for high-density arrays is the study of the variation in levels of gene expression between cell populations. Therefore, we synthesized two sets of gene expression microarrays, one set using the newly developed protocol for the reverse (5′→3′) synthesis, employing 3′-BzNPPOC phosphoramidites with a coupling time of 15 seconds and a photodeprotection with a radiant exposure of 3 J/cm^2^, whereas the other set was used for the forward (3′→5′) synthesis, carried out with 5′-BzNPPOC amidites but otherwise identical synthesis parameters. The gene expression microarray design consisted of a total number of 382536 oligonucleotides randomized across a checkerboard-like layout on the microarray surface and composed of human gene probes, reference sequences and quality controls. In detail, two replicates of each of at least 3 unique probes for more than 45000 human genes with a length of 60 nucleotides were synthesized, together with 20–100 replicates of 53mer and 60mer reference sequences and 25mer quality controls. Hybridization experiments with Cy3-labelled cDNA reverse-transcribed from mRNA extracted from a human colon adenocarcinoma cell line (Caco-2) were used to evaluate the quality of the forward and reverse microarrays. For each set of microarrays, one array was hybridized to a Cy3-labelled sample of the cDNA from untreated cells, serving as a control, whereas its mirror-image counterpart was hybridized to the Cy3-labelled cDNA from cells treated with cinnamaldehyde. The quality control metrics from the Cy3-labelled synthetic oligonucleotides (QC_25mer, EcoBioA1_53mer and EcoBioD2_60mer) are summarized in Table 2.

**Table 2:** Quality control data for labelled synthetic oligonucleotides used as hybridization quality controls for gene expression microarrays.

	3′→5′ synthesis		5′→3′ synthesis	
	***Average intensity***	***c***_***v***_	***Average intensity***	***c***_***v***_
QC-25mer	10460	0.098	11121	0.117
EcoBioA1	10509	0.106	10774	0.054
EcoBioD2	11219	0.079	11789	0.098
	***Expression***	***SE***	***Expression***	***SE***
QC-25mer	13403	0.98	13532	0.98
EcoBioA1	13164	0.97	12463	0.98
EcoBioD2	13568	0.97	14039	0.99

Average intensity shown in arbitrary units (a.u.) and coefficient of variation (c_v_) of non-normalized probe data (*top*) as well as average expression and standard error (SE) values of Robust Multi-array Average (RMA) normalized probe data (*bottom*).

**Figure 6:**
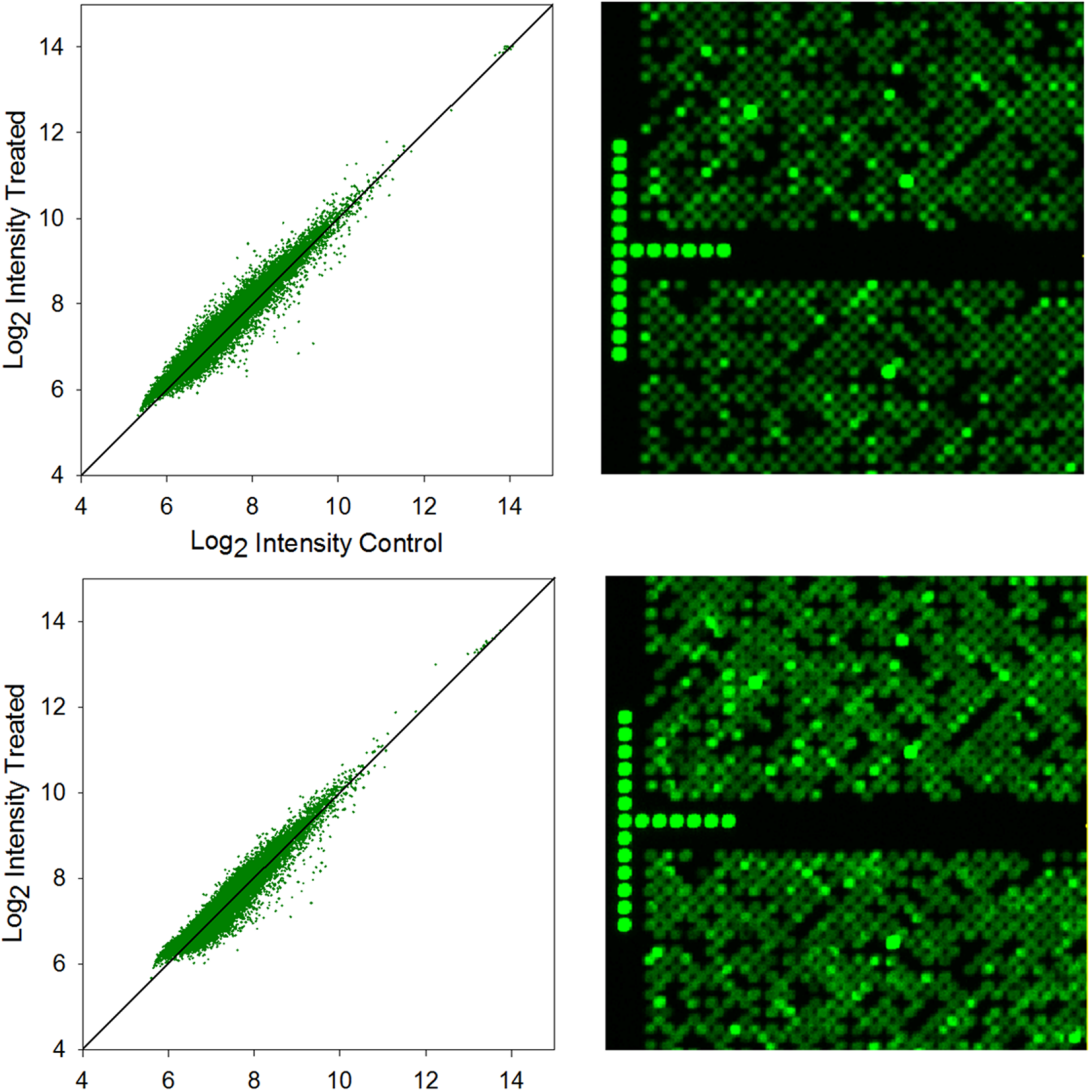
*Left*. Scatterplots of the RMA-processed data from the gene expression microarrays synthesized in the 3′→5′ (top) and in the 5′→3′ direction (bottom). Deviations from the diagonal line indicate differential gene expression. *Right*. Details of 2.5 µm resolution scan images from gene expression microarrays synthesized in the 3′→5′ (top) and in the 5′→3′ direction (bottom).

The normalized log_2_ transformed data are represented in fluorescence intensity scatter plots and shown in Fig. 6. The almost identical data distribution including deviations from the diagonal line, which represent differential gene expression patterns, together with the uniform quality control metrics (Table 2) indicate high quality and consistency in the fabrication of high-density gene expression microarrays, independent of the synthesis direction. This impression is enhanced by the very similar appearances of the scanned images of the hybridized microarrays (Fig. 6).

## Discussion

*In situ* synthesized DNA arrays are almost always synthesized in the traditional 3′→5′ direction, but important new applications of microarrays, such as spatial transcriptomics^3,24,25^ and enzymatic synthesis of RNA^40^, require a reverse, 5′→3ʹ oriented array synthesis or combined 5′→3′ and 3′→5′ synthesis. Our aim with this project was to adapt the well-established, phosphoramidite and cycle-based techniques for 3′→5′ *in situ* microarray fabrication to the efficient synthesis of high-density reverse (5′→3′) DNA microarrays of similarly high quality. The robust photolithographic method for high-density DNA array fabrication with up to 786432 individual sequences is being constantly improved and optimized, but those improvements only favoured the classical, 3′→5′ DNA synthesis^31^. While phosphoramidites with photolabile protecting groups at the 3′-OH position are commercially available, reverse array synthesis has been mostly overlooked. We therefore optimized the photolithographic *in situ* MAS system to the fabrication of 5′→3′ oligomers.

We first evaluated the coupling efficiency of phosphoramidites carrying BzNPPOC or NPPOC as photolabile protecting groups for the 3′-hydroxyl group. Even though previously published data indicated lower stepwise coupling yields for inverse NPPOC monomers than for their 5′-NPPOC counterparts^34^, we were able to obtain uniformly high coupling yields for 3′-NPPOC as well as for 3′-BzNPPOC amidites and their 5′-equivalents (Table 1). Since a shorter synthesis time results in improved microarray quality and allows for higher array throughput^31^, further investigations focused on shortening the coupling time of 3′-BzNPPOC or 3′-NPPOC phosphoramidites. Significantly reducing the coupling times (from the standard 60 seconds to 15 seconds) was found to improve synthesis quality, as indicated by the higher hybridization-based fluorescence intensities. An additional and meaningful reduction of the overall synthesis time is available through the use of 3′-BzNPPOC phosphoramidites. Indeed, further experiments focusing on determining the optimal UV light exposure for complete photolysis revealed that the BzNPPOC group is cleaved more than twice as fast as NPPOC.

Furthermore, we observed a 45% slower photodeprotection rate (photolytic efficiency; *εφ*_365nm_) for amidites carrying the photolabile group at the 3′-OH compared to 5′-OH protected equivalents (Fig. 3). The extinction coefficients at 365 nm for both the forward and reverse protected thymidine phosphoramidites are very similar (data not shown). The synthesis of microarrays using exposure gradients followed by hybridization is not a direct determination of photolytic efficiency and quantum yield, but these can be obtained by comparison with similar exposure gradient curves obtained for equivalent 5′-OH protected NPPOC derivatives with photolytic efficiencies and quantum yields known via direct methods, specifically, irradiation in solution followed by quantification of the photoproducts by HPLC^32^. For typical microarray applications, photodeprotection is regarded as complete when no significant improvement in hybridization signal is observed for higher exposures; this corresponds to ~95% photodeprotection. The resulting quantum yields for photodeprotection of the 3′-BzNPPOC and 3′-NPPOC phosphoramidites are significantly lower, 0.46 and 0.22, respectively, than their 5′ equivalents^32^.

The photolysis of *o*-alkyl-nitrophenyl photolabile protecting groups proceeds via a β-elimination reaction^41,42^. Upon absorption of a photon, the excited nitro group abstracts the intramolecular hydrogen leading to the formation of the transient *aci*-nitro species. The decay of the *aci*-nitro intermediate strongly depends on factors such as substitutions, pH and leaving group identity. For good leaving groups, the product release is assumed to be synchronous with the decay of the *aci*-nitro intermediate, whereas it might be rate-limiting for poor leaving groups^42–44^. The slower rate of uncaging may be ascribed to the nature of the resulting hydroxyl group, with the 3′ hydroxyl being released slower than the 5′ hydroxyl, and may originate from the leaving group ability of a secondary alcohol, or its carbonate equivalent.

We also found that the optimal deprotection time depends on the protecting group strategy for the exocyclic amine of the nucleobase. Fast-deprotecting groups, like Pac, *t*BPac and *i*PrPac, are fully removed after a 2 hour exposure in an EDA/EtOH (1:1, v/v) solution, while standard protecting groups, e.g. Ac, Bz, *i*Bu, require longer deprotection periods. However, the deprotecting conditions are sufficiently mild to allow overnight deprotection without any degradation of the microarray surface. Microarray deprotection being an important factor in synthesis throughput, the use of faster alternative deprotection conditions, for example aqueous methylamine^45^, is an alluring prospect but hinges on how the deprotection solutions affect the DNA-glass attachment chemistry, which is beyond the scope of this paper. These improvements now allow for the fabrication of reverse-oriented DNA oligonucleotides by photolithography that rivals the quality of the already optimized, standard 3′→5′ DNA array synthesis. We explored the promising applications of the established reverse synthesis protocol in highly complex and demanding array designs by fabricating a high-density gene expression microarray and hybridizing it to Cy3-labelled genomic cDNA. The comparable fluorescence intensities for 3′→5′ and 5′→3′ oriented gene expression microarrays further reinforce the accuracy and efficiency of light-directed reverse DNA synthesis while the overall distribution of fluorescence values (see Fig. 6) suggests that forward and reverse arrays equally discriminate against mismatches. The application of the newly established protocol for the synthesis of fully deprotected DNA oligonucleotides oriented in the reverse (5′→3′) direction on a solid surface in high yield can especially be of great value in a highly flexible synthesis setup that enables the parallel synthesis of both forward (3′→5′) and reverse (5′→3′) oligonucleotides on the same surface. This not only allows for the fabrication of pure forward and reverse oligonucleotides on microarrays, but also for their combination in single strands which in turn opens up the possibility for inclusions of segments functioning, for example, as regions with increased nuclease resistance, or the synthesis of microarrays of complex DNA architectures.

## Conclusion

Here we report on a method for a highly efficient, high-density photolithographic *in situ* synthesis of fully deprotected, reverse (5′→3′) DNA oligonucleotides on microarrays using commercially available phosphoramidites with 3′-OH photolabile protecting groups as building blocks. Careful optimization of synthesis parameters allowed for the minimization of synthesis time without sacrificing on the quality of the resulting microarrays. Together with our synthesis setup, this method offers a highly flexible design of individual DNA strands, opening up the possibility for combining forward and reverse oriented segments in single strands and the complete *in situ* synthesis of double-stranded DNA arrays.

## Methods

### Substrate preparation

All microarrays were simultaneously synthesized as mirror image pairs according to a method published earlier^46^. In order to enable access to the synthesis area in-between the two glass substrates (Schott Nexterion Glass D microscope slides; 75 × 25 × 1 mm), the bottom slide was drilled with two holes of approximately 1 mm diameter. Prior to functionalization, both top and bottom slides were cleaned in an ultrasonic bath and rinsed with purified water. The glass substrates were silanized with *N*-(3-triethoxysilylpropyl)-4-hydroxybutyramide (Gelest SIT8189.5) in a 95:5 (v/v) solution of ethanol/water containing 0.2% acetic acid for a period of 4 hours at room temperature under gentle agitation^47^. After two washing steps for 20 minutes each in a 95:5 (v/v) EtOH/H_2_O + 0.2% AcOH solution, the slides were dried and cured in a vacuum oven overnight at 120 °C. The functionalized slides were stored in a desiccator until use.

### Microarray synthesis and deprotection

The fabrication of microarrays as mirror image pairs was performed as already published^46^ using a Maskless Array Synthesizer (MAS) instrument which links an optical system with a synchronized chemical delivery system. The computer-controlled optical system uses a digital micromirror device (Texas Instruments 0.7 XGA DMD) consisting of an array of 1024 × 768 individually addressable mirrors, replacing the need for physical photomasks, to pattern UV light onto the synthesis area. A high-power 365 nm UV LED (Nichia NVSU333A U365 surface-mount LED) serves as light source^48^. The UV light intensity reaching the synthesis area is adjusted using a calibrated intensity meter (SÜSS Micro-Tec 1000). The computer-controlled light exposures which trigger the removal of the photolabile protecting groups are synchronized with a chemical fluidics system (Expedite 8909 nucleic acid synthesizer), which delivers solvents and reagents to the synthesis area. The phosphoramidite chemistry is similar to that of standard solid-phase oligonucleotide synthesis, except for the use of photolabile protecting groups that can be removed upon absorption of a near UV photon in the presence of a weak amine base (here, 1% imidazole in DMSO), leaving either the 5′- or the 3′-hydroxyl group available for the following coupling with an activated monomer. After the synthesis, the phosphodiester and the nucleobase protecting groups are removed by exposing the microarrays to a solution of 1:1 (v/v) ethylenediamine/ethanol for 2 or 12 hours at room temperature. Subsequently, the microarrays are rinsed with deionized water and dried using a microarray centrifuge.

### BzNPPOC and NPPOC phosphoramidites

BzNPPOC and reverse BzNPPOC DNA phosphoramidites were purchased from Orgentis. NPPOC and reverse NPPOC DNA phosphoramidites were obtained from ChemGenes. All phosphoramidites were diluted to 30 mM with acetonitrile (<30 ppm water; Sigma-Aldrich). The protecting groups for the exocyclic amines of the nucleobases are shown in Table 3.

**Table 3:** Protecting groups for the exocyclic amines of the phosphoramidite nucleobases.

	dA	dC	dG
5′-NPPOC	*t*BPac	*i*Bu	*i*PrPac
5′-BzNPPOC	*t*BPac	Ac	*t*BPac
3′-NPPOC	*t*BPac	*i*Bu	*i*PrPac
3′-BzNPPOC	Bz	Ac	*i*Bu

### Coupling efficiency determination

The yield of each coupling step was determined by the method of terminal labelling as previously described^35^. Microarrays were synthesized with DNA strands of various lengths from 1 to 12mers. To prevent failed sequence additions, each coupling was followed by a capping step with 5′-DMTr-dT (0.03 M) for 60 seconds. Features that were used to determine the background fluorescence remained unlabelled. The other features were terminally labelled by performing two consecutive coupling steps of 300 seconds with Cy3 phosphoramidite (0.05 M). After completion of the synthesis, the microarrays were washed in acetonitrile at room temperature for a period of two hours and dried with a microarray centrifuge. The arrays were scanned as described below and the data was extracted with NimbleScan. SigmaPlot 11.0 (Systat Software) was used to normalize and plot the data. The mathematical model of an exponential decay can be used to describe the decrease in fluorescence intensity as a function of the increasing length of the sequence. The resulting curve was fit to the model curve *y *= *ae*^−*bx*^ with *y* being the fluorescence intensity, *a* the initial intensity, *x* the number of couplings and *1 *− *b* the fractional stepwise coupling yield.

### Coupling time optimization

The coupling experiments were carried out using the synthesis protocol shown in Table 4, with different coupling times, ranging from 15 to 60 seconds (15, 30, 45 and 60 s).

**Table 4:** Representative chemical synthesis protocol, in Expedite 8909 format, used for the synthesis with BzNPPOC phosphoramidites.

Cycle BzNPPOC-dA				
Function	Mode	Pulses	Sec	Description
**$Coupling**				
1/*Wsh	Pulse	20	0	Flush with Wsh
2/*Act	Pulse	6	0	Act
18/*A + Act	Pulse	9	0	A + Act
2/*Act	Pulse	6	0	Push with Act
1/*Wsh	Pulse	3	***X***	Couple monomer
1/*Wsh	Pulse	10	0	Flush with Wsh
**$Capping**				
40/*Gas A	Pulse	1	10	Dry column
**$Oxidizing**				
15/*Ox	Pulse	3	0	Ox to column
12/*Wsh A	Pulse	10	0	Flush with Wsh A
17/*Aux	Pulse	15	0	Exposure solvent
130/*Event 2 Out	NA	4	3	Event 2 out
17/*Aux	Pulse	10	25	Exposure solvent
12/*Wsh A	Pulse	5	5	Flush with Wsh A
130/*Event 2 Out	NA	4	3	Event 2 out
12/*Wsh A	Pulse	15	0	Flush with Wsh A

The coupling time ***X*** was set to 15, 30, 45 or 60 seconds.

### Photolysis efficiency determination

To examine the photolysis efficiency and determine optimal light exposure parameters for the removal of the photocleavable protecting groups, exposure gradient experiments were performed according to a previously published method^32^. The corresponding microarrays were designed with a light-exposure gradient. In this design, 30 virtual masks are displayed on the DMD within the timeframe of a single, complete exposure step. Each of the virtual masks was displayed for 1/30^th^ of the time required to reach the maximum exposure time necessary for each experiment. The first mask enables the deprotection of phosphoramidites on all features of the microarray where DNA strands are being synthesized. Each of the subsequent masks exposes fewer areas of the microarray in order to obtain oligonucleotide probes which were deprotected with the maximum exposure as well as probes deprotected with successively lower UV light exposure. 65 replicate probes were synthesized at each of the 30 exposure levels with an exposed feature size of 70 × 70 µm, corresponding to a 5 × 5 array of DMD mirrors, and their location randomized across the microarray surface.

### Consecutive synthesis of DNA strands

In order to be able to make direct and reliable comparisons of DNA strands synthesized in different directions or with various coupling times, oligonucleotides need to be synthesized on the same microarray substrate. The desired sequences are randomized across the microarray surface and are fabricated consecutively. Oligonucleotide 25mer probes synthesized early show a ∼6% reduced hybridization intensity, in comparison with the next probe synthesized, due to chemical damage caused by prolonged exposure to solvents and reagents.

### Comparison of forward and reverse synthesis

Forward synthesis describes the fabrication of DNA strands in the 3′ to 5′ direction, whereas the reverse synthesis occurs in the 5′ to 3′ direction. In order to make direct comparisons between forward and reverse syntheses, both were performed on the same substrate. Therefore, the microarray was designed for a consecutive synthesis of the desired sequences, instead of the standard parallel synthesis of the DNA strands. The consecutive synthesis started with the fabrication of a forward sequence, followed by reverse synthesis of the same sequence and a repetition of both syntheses as shown below:

3′-TTTTTCTGGTCCCACCAAGTACTACTACTGtttttgtcatcatcatgaaccaccctggtcTTTTTCTGGTCCCACCAAGTAC TACTACTGtttttgtcatcatcatgaaccaccctggtc

A, C, G, T: forward, 5′-photoprotected phosphoramidites

a, c, g, t: reverse, 3′-photoprotected phosphoramidites

The location of the probe replicates of the sequences were randomized across the microarray surface.

### Genomic cDNA

The human colon carcinoma Caco-2 cell line (ATCC) was cultivated under humidified atmosphere at 37 °C and 5% CO_2_ in Dulbecco’s modified Eagle medium (DMEM) with addition of 10% fetal bovine serum, 4 mM L-glutamine and 1% penicillin/streptomycin^49^. After having been grown to 80–90% confluence, cells were seeded in 6-well plates at approximately 4 × 10^5^ cells/9.6 cm^2^ and maintained for 21 days to allow differentiation to an enterocyte-like phenotype. Cultivation medium was changed every second to third day. For the microarray experiments, a previously published protocol was used^50^. Briefly, cells were treated with DMEM devoid of fetal bovine serum with or without 1000 µM of the test compound, cinnamaldehyde, for 3 hours on day 21. After subsequently washing the Caco-2 cells with ice-cold PBS, RNA isolation was performed using the RNeasy Mini Kit (Qiagen). Concentration and quality of the isolated RNA was determined photometrically using a NanoQuant Plate on an Infinite M200 Tecan reader prior to reverse transcription of a total of 10 µg RNA using Cy3-labelled random nonamer primers (Tebu Bio) as described by Ouellet *et al*.^51^. Cy3-labelled cDNA was then purified by means of a Qiagen Qiaquick PCR purification kit.

### Microarray hybridization

The non-genomic microarrays were hybridized to Cy3-labelled complementary oligonucleotides (IDT or Eurogentec). Deprotected microarrays were hybridized in a self-adhesive hybridization chamber (Grace Biolabs SA200). The hybridization mix consists of 150 µL 2x MES hybridization buffer, 110 µL nuclease free water, 13.3 µL acetylated BSA (10 mg/mL) and 26.7 µL of the 100 nM labelled complementary oligonucleotides. The microarrays were incubated with rotation for a period of 2 hours in a hybridization oven (Boekel Scientific) at 42 °C. The hybridization chamber was then removed and the microarrays were washed in non-stringent wash buffer (SSPE; 0.9 M NaCl, 0.06 M phosphate, 6 mM EDTA, 0.01% Tween20) for 2 minutes, in stringent wash buffer (100 mM MES, 0.1 M NaCl, 0.01% Tween20) for 1 minute, followed by a short wash in final wash buffer (0.1x SSC) for a few seconds. A microarray centrifuge was used to dry the microarrays.

In most of the performed experiments, the Cy3-labelled complimentary oligonucleotide referred to the following sequence: 5′-GAC CAG GGT GGT TCA TGA TGA TGA C-3′, (QC_25mer). The microarrays used for comparison experiments of forward and reverse synthesized oligonucleotide strands were hybridized to a 1:1 mix of 5′-Cy3 and modified 3′-Cy3 labelled complementary oligonucleotides. The modification of the 3′-Cy3-QC 25mer refers to the presence of a C- instead of a G-base at the 3′-end. Therefore, the sequence of the 3′-Cy3-labelled 25mer oligonucleotide is as follows: 5′-GAC CAG GGT GGT TCA TGA TGA TGA G-3′ (modified QC_25mer).

### Gene expression microarray hybridization quality control

The hybridization solution used for gene expression experiments consisted of 135 µL 2x MES hybridization buffer, 15 µL acetylated BSA (10 mg/mL), 3 µL herring sperm DNA (10 mg/mL), 10 µL Cy3-labelled QC_25mer (100 nM), 10 µL Cy3-labelled ECO1BioA1 (100 nM), 10 µL Cy3-labelled ECO1BioD2 (100 nM) and the Cy3-labelled cDNA in 85 µL water. The sequences of the 5′-Cy3-labelled oligonucleotides used to evaluate the synthesis and hybridization quality of the microarrays are shown below (5′ to 3′):

- GAC CAG GGT GGT TCA TGA TGA TGA C, QC_25mer
- GAT TTA GGT TTA CAA GTC TAC ACC GAA TTA ACA ACA AAA AAC ACG TTT TGG AG, ECO1BioA1_53mer
- GAA ATG AGG GTG TAA TTG ATT GGG CAA CTG TGC GCC ACG CTA CTT TCT TCT TCG CTT AAC, ECO1BioD2_60mer

The gene expression microarray design consisted of 100 probes for QC_25mer, and 140 probes for each of the ECOBio-quality control sequences. The location of each microarray feature was randomized along with all other probe sequences. The gene expression microarrays were incubated with rotation for a period of 20 hours in a hybridization oven (Boekel Scientific) at 42 °C, then washed and dried as described above.

### Data analysis and microarray quality control

A microarray scanner (GenePix 4100 A, Molecular Devices) was used to scan the dried hybridized microarrays at 5 µm resolution with an excitation wavelength of 532 nm. Data extraction of the scanned images was performed with the Software NimbleScan 2.1 (Roche NimbleGen).

### Gene expression data analysis and microarray quality control

A microarray scanner (GenePix 4400 A, Molecular Devices) was used to scan the dried hybridized gene expression microarrays at 2.5 µm resolution with an excitation wavelength of 532 nm. Data extraction of the scanned images was performed with the software NimbleScan 2.1 (Roche NimbleGen). The robust multichip analysis (RMA) function was used to normalize the extracted probe-level data. The normalized intensities of the two biological datasets were log_2_ transformed and plotted as treated *versus* control.

## Data Availability

The datasets used and/or analysed during the current study are available from the corresponding author on reasonable request.
